# Multi-Omics Analysis in Initiation and Progression of Meningiomas: From Pathogenesis to Diagnosis

**DOI:** 10.3389/fonc.2020.01491

**Published:** 2020-08-28

**Authors:** Jiachen Liu, Congcong Xia, Gaiqing Wang

**Affiliations:** ^1^Clinical Medicine, Xiangya Medical College of Central South University, Changsha, China; ^2^Department of Neurology, Sanya Central Hospital (The Third People's Hospital of Hainan Province), Sanya, China

**Keywords:** meningiomas, biomarker, genomics, epigenomics, transcriptomics, proteomics

## Abstract

Meningiomas are common intracranial tumors that can be cured by surgical resection in most cases. However, the most disconcerting is high-grade meningiomas, which frequently recur despite initial successful treatment, eventually conferring poor prognosis. Therefore, the early diagnosis and classification of meningioma is necessary for the subsequent intervention and an improved prognosis. A growing body of evidence demonstrates the potential of multi-omics study (including genomics, transcriptomics, epigenomics, proteomics) for meningioma diagnosis and mechanistic links to potential pathological mechanism. This thesis addresses a neglected aspect of recent advances in the field of meningiomas at multiple omics levels, highlighting that the integration of multi-omics can reveal the mechanism of meningiomas, which provides a timely and necessary scientific basis for the treatment of meningiomas.

## Introduction

Meningiomas account for 13–36.6% of the primary malignant tumors of the central nervous system (1). Although the reported incidence is around 7.8/100,000 (2), the rate of recurrence increases dramatically to 32% with progressive/higher grade meningiomas (~20% of all meningiomas) (3). Coupled with the high treatment costs (~$83,838 per person) (4), meningioma is increasingly recognized as a serious, worldwide public health concern (5). Since the publication of revised WHO guidelines in 2016, the diagnosis of meningioma is mainly divided into three grades based on the morphological features (6). Unfortunately, this grading system does not ultimately predict the clinical behavior of meningiomas, especially long-term recurrence of atypical meningiomas (7).

**Figure 1:**
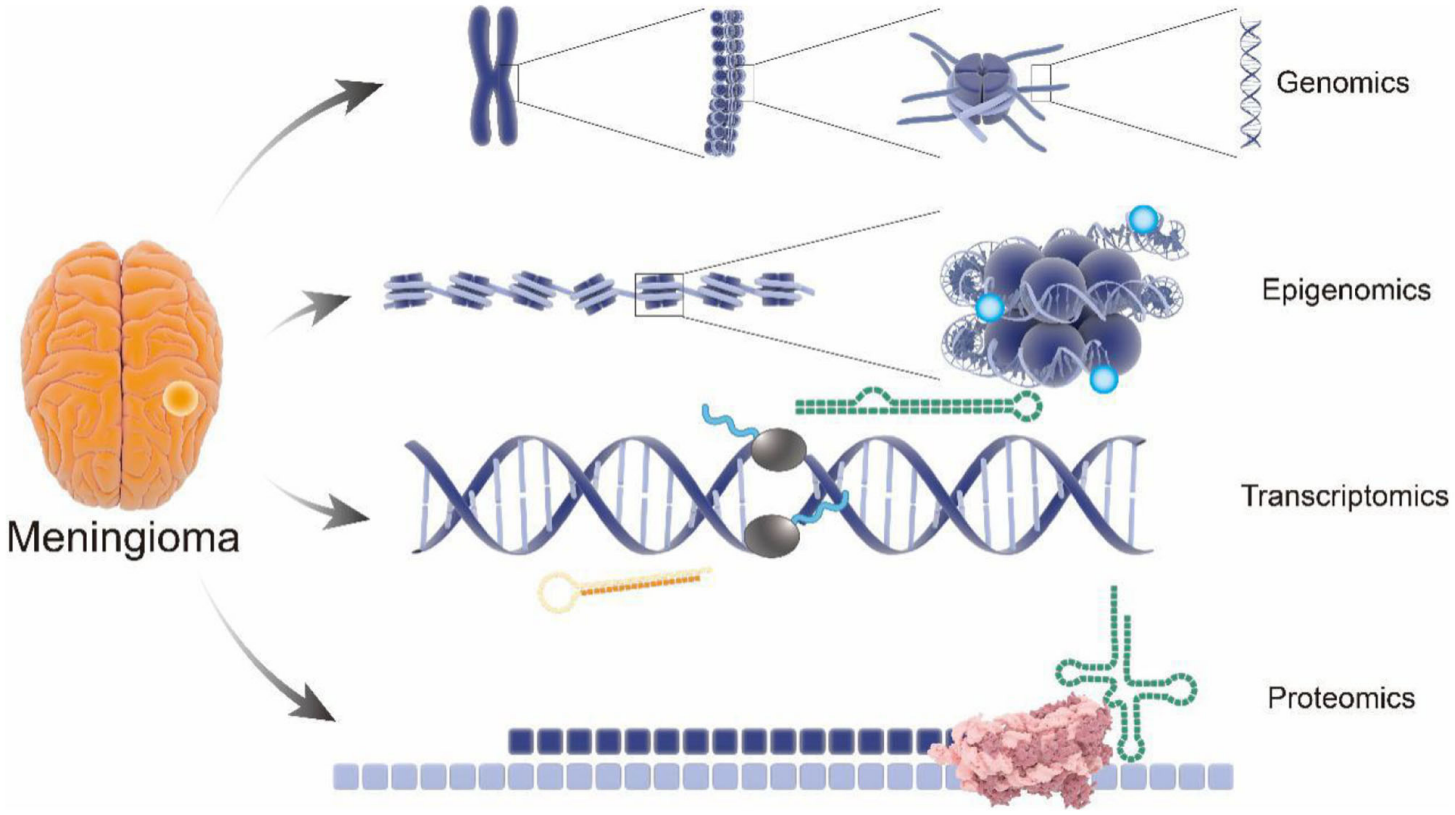
Comprehensive analysis of pathology and molecular genetics of meningioma from multi-omics perspective. Structure of gene with the meningioma pathogenic variants have been revealed by genomics; performance of the epigenomics showing the influence of the genetic modification on meningioma; pathological gene expression in meningioma were analyzed by transcriptomics; applications of proteomics visually show the endocranial shape changes during meningioma. From genomics to proteomics, the pathological process and potential therapeutic targets involved in meningioma progression will be revealed as never before.

Recent advances in omics technologies (genomics, transcriptomics, epigenomics, and proteomics) contribute to large screening of biomarkers for meningioma by tissue microarray to predict biological behavior of meningiomas (8). Notably, integration of multi-omics with clinical data represents an accurate and promising methodology to provide very accurate prediction models for meningioma progression (Figure 1), suggesting the potential of early and accurate diagnosis, effective therapeutic strategies, and favorable prognosis of meningioma (9, 10).

## Genomics

Accurate and comprehensive sequencing of personal genomes is an important technical advance based on bioinformatics analysis (11), which is crucial to genetic studies of complex human diseases (12). Deep understanding of genetic alterations relating to meningioma development and progression may provide new insights into meningioma classification and personalized treatment (13).

**Figure 2:**
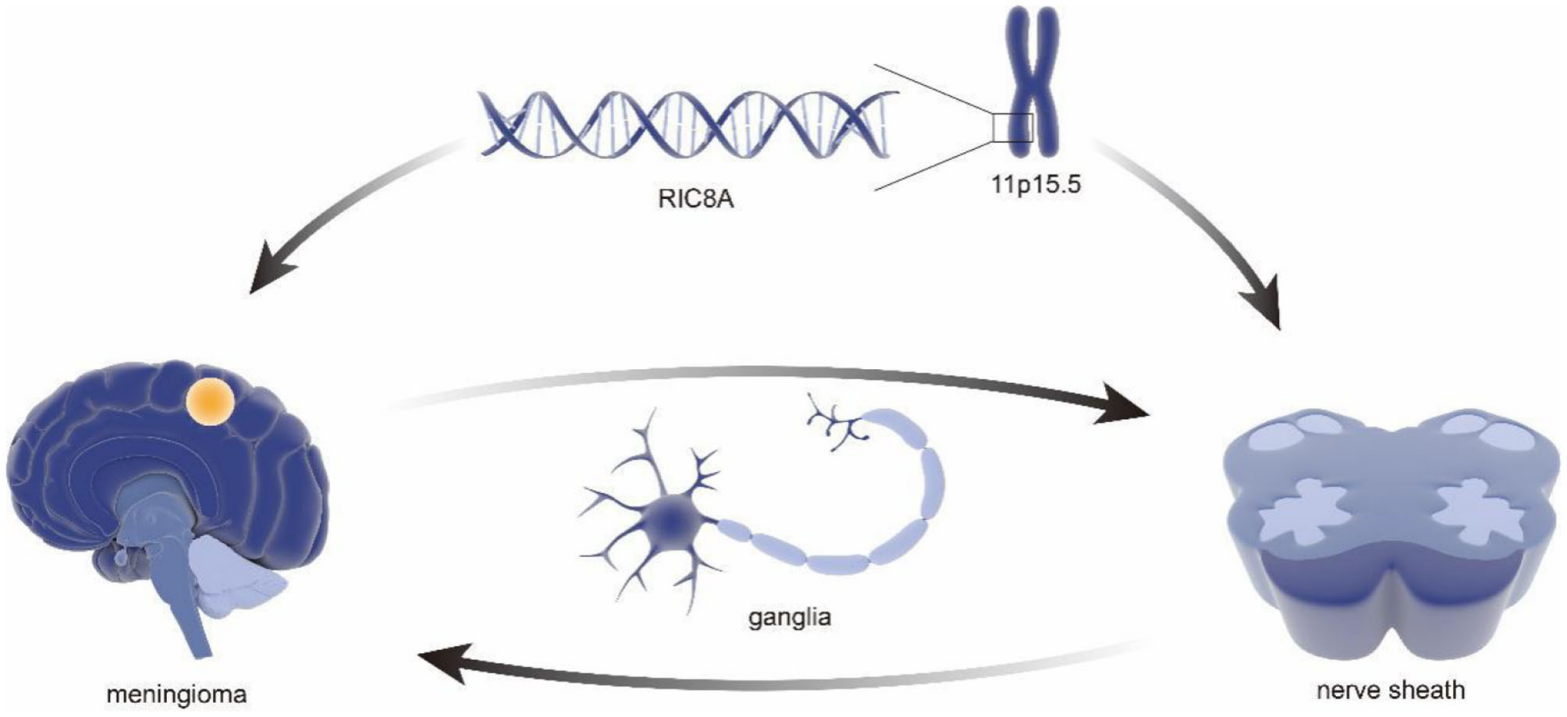
Genetic association of the nerve sheath development and meningioma. The RIC8A located in area 11p15.5 were revealed to be associated with pathological phenotypes in meningioma. It is important to mention that the same genes have been confirmed to be related to cranial neural crest-derived structures. Consider the correlation between nerve sheath and ganglia, which might explain a series of cases of nerve sheath meningioma and ganglia intraparenchymal meningioma.

As early as 2011, a sequencing-based genome-wide association study (GWAS) of 859 patients with meningioma and a control group (*n* = 704) identified MLLT10 as a new susceptibility locus (14). It is worth mentioning that, in the last decades, the role of *MLLT10* in the pathogenesis and progression of meningioma has been well-established (15, 16). In that same year, an expanded genome-wide association study of meningioma, including 2,000 patients and 6,000 controls, was initiated by the National Institutes of Health (17), which earned a significant contribution in understanding genetic factors of meningioma. Notably, the results, including an inverse relationship between hormones and allergies, provided a clear framework and direction for further meningioma study as well as the establishment of comparative oncology (18–20). Furthermore, a genotype analysis in 65 samples using high-density single nucleotide polymorphism (SNP) arrays found associations between meningiomas and variation in *PIAS2, KATNAL2, TCEB3C, TCEB3CL*, and *CTNNA3*, especially *TARDBP* mutations with amyotrophic lateral sclerosis (21), which further improves the identification of susceptible sites of meningioma by genomics. Subsequently, a GWAS involving 1,606 meningioma patients and 9,823 controls provided additional support for the link between obesity and risk of recurrence in meningioma (22), which laid a solid foundation for meningioma characteristics, including risk factors and epidemiology (23, 24). To further illustrate the genetic basis and construct a genetic linkage map of meningioma, Claus et al. identified a new meningioma susceptibility site at 11p15.5 through a combined reference panel from UK10K data including a total of 2,138 and 12,081 controls and 1,000 genomic projects in 2018 (25). It is worth pointing out that the susceptible site included a new pathogenic mutation in *RIC8A*, which is necessary for the development of cranial neural crest-derived structures. Therefore, this study suggests the cytogenetic relationship between meningiomas and nerve sheath structures (26) (Figure 2).

In addition to the potential role of genetic factors on meningioma, genomics has been applied in the diagnosis and classification of meningioma. Clinically, in the case of a meningioma specimen that contains atypical tumor regions that are difficult to assess, molecular marker techniques for patient genome analysis, such as array comparative genomic hybridization (aCGH) and expression array profiles, can be used for histopathological grading (27). The first instance from a whole genome sequencing project of malignant subtypes revealed mutational signatures and frequently altered genes in malignant meningiomas, including *NF2, MN1, ARID1B, SEMA4D*, and *MUC2*, which confirmed the role of pathogenic *NF2* mutations in the development of meningiomas, and expression of *MN1* may be a valuable diagnostic tool for determining the potential in malignant transformation (28).

So far, genomics has made a tremendous contribution to the criteria for diagnosis, staging, risk stratification, and response assessment of meningiomas (Table 1). Regrettably, some aspects of genetic factors in meningioma have been ignored, and the gene regulatory network leading to meningioma remains unclear. Further pooling research in genomics will advance the field of meningiomas' genetic basis and pathological mechanisms, which may also provide novel research horizons and suggestions for intervention strategies and clinical practice of meningioma.

**Table 1:** Genomics research associated with meningiomas.

**Phenotype**	**Sample size (case, control)**	**Tissue/tissue**	**Ethnicity**	**Tested genes/techniques**	**Major results**	**PMID**
Meningiomas and normal	*N* = 1,563	Meningiomas tissue (I, II, III)	German	10p12.31, MLLT10	Marker of meningiomas with WHO grade I, II, III	21804547(14)
Meningiomas and normal	*N =* 14,219	Meningiomas tissue (I, II, III)	German	RIC8A	Marker of meningiomas with WHO grade I, II, III	29762745(25)
Meningiomas	*N =* 9	Meningiomas tissue (III)	China	NF2	Important marker of meningiomas with WHO grade III	25549701(28)
Meningiomas	*N =* 9	Meningiomas tissue (III)	China	MN1	Candidate gene for malignant transformation of meningioma	25549701(28)
Meningiomas	*N =* 9	Meningiomas tissue (III)	China	ARID1B	Marker of meningiomas with WHO grade III	25549701(28)
Meningiomas	*N =* 9	Meningiomas tissue (III)	China	SEMA4D	Marker of meningiomas with WHO grade III	25549701(28)
Meningiomas	*N =* 9	Meningiomas tissue (III)	China	MUC2	Marker of meningiomas with WHO grade III	25549701(28)

## Epigenomics

Epigenetic factors, mainly DNA methylation and histone modification, have considerable effects on the pathogenesis of meningioma (29). In the last few years, developments in multi-omics technologies provide tools for high-throughput and high-density molecular analyses, which has provided a novel view regarding the functional organization of the molecular layer. The pathogenic role of chromosome markers in gene regulation and other processes were also inferred by it (30).

WHO classification of meningiomas is based on histologic characteristics. However, part of malignant meningiomas was histologically described to benign meningiomas (31); therefore, novel diagnostic strategies are urgently required while DNA methylation assessment has considerable potential to reconstruct the grade of meningioma. Expression profiles of 10,422 genes at the early stage of meningioma using cDNA microarray indicate hypermethylation of gene subsets are critical in tumor development (32). Further research identified 64-CpG meningioma methylation predictor (64-MMP), which is responsible for tumor recurrence (hazard ratio = 12.16) (33). In 2017, Sahm et al. compiled a genome-wide mapping of differentially methylated regions by DNA methylation profiling from 497 meningioma and 309 extra-axial skull tumors that might histologically mimic meningioma variants. On this basis, six different clinically relevant methylation types of meningioma were distinguished, and they relate to typical mutations, cytogenetics, and gene expression patterns (34). Notably, the classification by methylation provides more precise prognostication of progression-free survival outcomes at 10 years' follow-up compared to WHO grading, which highlights the diagnostic and prognostic implications of malignant meningioma by assessing methylation status.

Importantly, epigenetic profiles in meningioma contribute to the construction of an individualized prediction model of early progression and recurrence in meningioma (35). For example, DNA methylation profiles of 282 clinically annotated meningioma samples were used for construction of a prediction model of 5-year recurrence-free survival (RFS) in meningioma. Notably, the recurrence model provides important prognostic information (hazard ratio = 7.7, area under curve = 0.82), which is more accurate than prediction based on clinical factors, including extent of resection and WHO grade (Δ area under curve = 0.25) (36).

In addition to the roles in tumor classification, a comprehensive understanding of epigenetic regulation that has characterized meningioma development and progression may also provide useful guidance for targeted therapies. So far, methylation of *TIMP3, CDKN2*, and other genes that can regulate the progression of meningiomas have been identified by genome-wide methylation DNA analysis (37); further work reveals the connection between the H3K27me3 signal and hypermethylated phenotype in meningiomas, integrating with microarray analysis of the transcriptional network controlled by *E2F2* and *FOXM1*. This study makes recommendations for potential targets for therapeutic intervention (38).

The progress in epigenetic research on meningioma have proved to be a valuable tool in pathological classification and intervention of meningiomas (Supplementary Table 1) (21). However, recent advances in epigenomics of meningioma have mainly focused on DNA methylation; the role of histone modification and chromosome organization have been neglected. It is also worth noting that chromosomes are associated with homologous recombination repair (HRR) defects, which has been confirmed as a primary causative factor of meningioma (39, 40), suggesting that histone modification has great potential in the development of novel meningioma prevention and intervention measures.

## Transcriptomics

By comparing the transcriptome differences between meningioma patients and controls, transcriptomics can screen out the specific expression differences with diagnostic significance, which can be used in the diagnosis and early intervention of meningiomas.

Since the occurrence and development of meningiomas are often caused by the accumulation of multiple gene changes, transcriptome can detect the gene expression differences between normal tissues and meningiomas from the transcriptional level (Supplementary Table 2) (41–45). In 2017, a genome-wide array comparing microRNAs expression in meningioma from 50 patients showed that miRNA-21 expression increased significantly with increasing histopathologic grade with reduction of miRNA-107 (41, 46). Notably, upregulated miR-29c-3p coupled with reduction of its predicted target recombinant pentraxin 3(*PTX3*) was observed in the same year using whole transcriptome microarray chips, which indicated the level of tumor suppressor *PTX3* is inhibited by miR-29c-3p (42). Interestingly, *PTX3* overexpression was frequently observed in high-grade gliomas and meningiomas with poor prognosis, which suggests that *PTX3* may be an important contributor to meningioma cell proliferation and invasion (47). The conflicting results have been obtained, which remind us that further studies of changes in transcriptome of meningioma is necessary.

As mentioned earlier, due to its high recurrence rate and poor prognosis, a lot of work on the research of malignant meningioma is required (48), and it is associated with shorter progression-free and overall survival after complete resection (49). Fortunately, novel markers of malignant meningiomas identified through differential gene expression analyses can be achieved through transcriptomics. For example, an illumina expression microarray to assess gene expression levels from a sample set of 19 resected meningiomas identified dense coexpression subnetworks in meningioma and detected carcinogenic modules associated with malignant meningioma. Among the 23 identified coexpression modules, a module involving 356 genes is highly correlated with occurrence of meningioma. It should be noted that putative meningioma tumor suppressive meningioma 1 (MN1) in this module was differentially expressed between malignant and benign meningioma (43), indicating it can be used as a predictor of meningioma classification.

**Figure 3:**
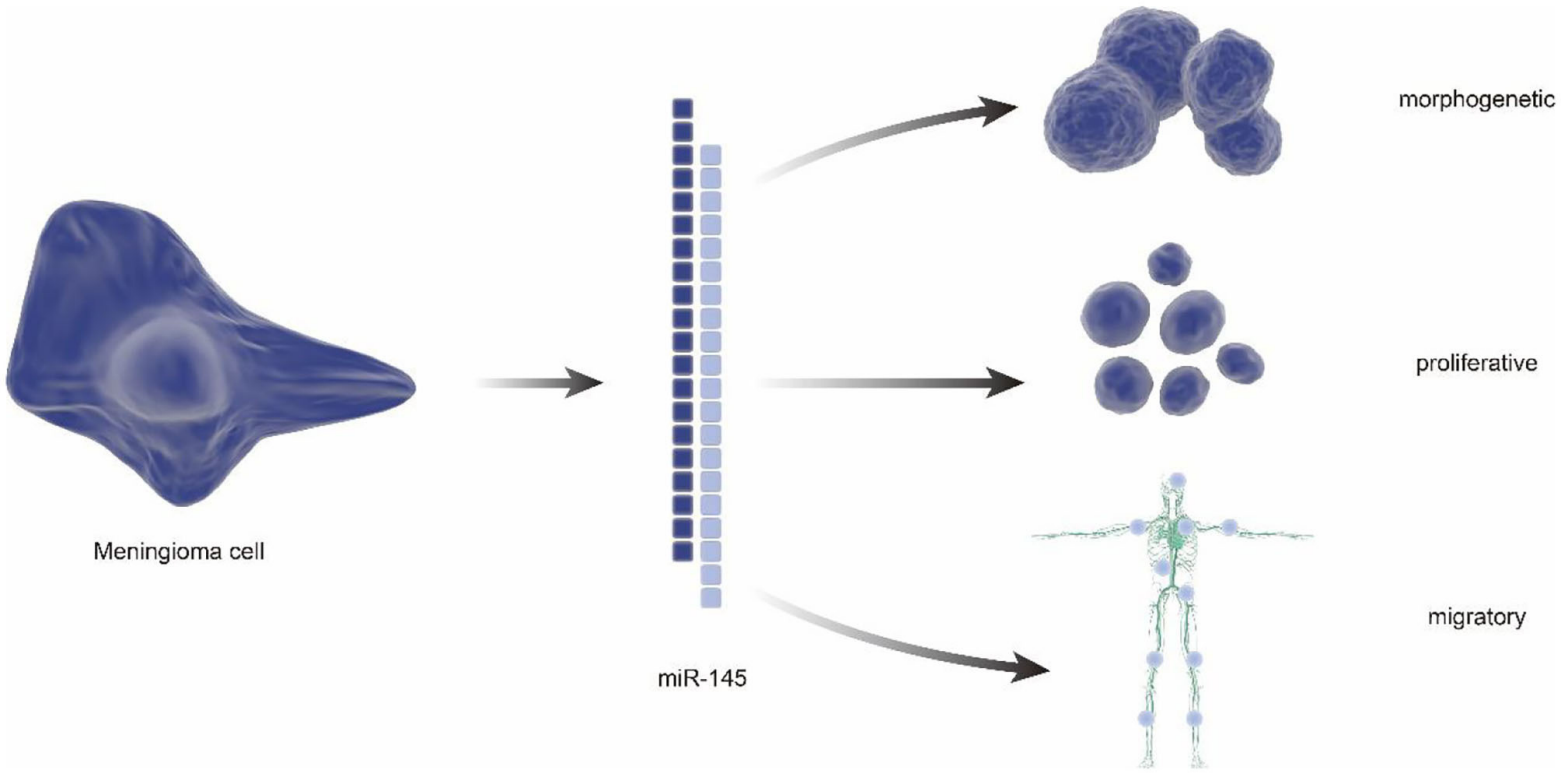
The regulatory role of miRNA in meningiomas. miRNA has a reduced expression in atypical and anaplastic meningiomas, which increases cell proliferation and reduces apoptotic susceptibility. In addition, reduced miRNA reduces migration, invasion, and adhesion of meningioma cells. It can also alter meningioma cell morphology, resulting in low elongation and adhesion.

In addition to characterization of differentially expressed genes, some RNAs were also found to have potential meaning in classification of benign and malignant meningiomas. In 2013, a tissue microarray indicated reduced expression of miR-145 in WHO grade II/III meningiomas using frozen samples from 42 meningiomas. Notably, the follow-up studies demonstrated the antiproliferation, morphogenesis, and antimigration effects of miR-145 in meningioma cells, suggesting the proposed role of the miR-145 in restraining meningioma progression (44) (Figure 3). Besides, the small nucleolar RNAs(snoRNAs), such as SNORA46 and SNORA48, were also found differentially expressed between grade I and grade II/III meningiomas, which is identified by RNA sequencing (RNA-seq) analysis after numerous genes were found differentially expressed by real time-PCR (45).

In addition, miRNAs belong to small ncRNAs (sncRNAs), and small interfering RNA (siRNAs) are functionally similar to miRNA, modulating post-transcriptional gene expression by binding to specific mRNAs (50). But transcriptomics studies focused on miRNAs are much more than that on siRNAs although siRNA has been found to relate to some meaningful molecules in meningiomas. For example, siRNA can decrease the expression of high-mobility group nucleosome-binding protein 5 (HMGN5), which has a positive association with meningioma histological grade (51). As for snoRNA, more and more evidence reveal the importance of snoRNA in tumorigenesis (52, 53), such as SNORD50A/B (C/D box), which can directly bind to and inhibit K-rat sarcoma (K-Ras), is deleted in many cancer types (54). However, the lack of transcriptomic studies pertaining to the expression of siRNAs and snoRNAs or relative pathways suggest that transcriptomic studies taking siRNA into consideration are required in the field of meningioma research.

## Proteomics

Proteomics is a large-scale study of protein properties, including protein expression levels, post translation modification, protein–protein interaction, etc., which has been proven to be a useful tool in the identification between varieties of meningeal neoplasms (55).

Proteomics can detect the differential expression of proteins in different grades or types of meningiomas (Supplementary Table 3) (56–58). As early as 2006, the pure meningioma cell population was sequenced to indicate the differentially expressed proteins of each WHO grade meningioma. This study identified the 15 proteins that were significantly related to atypical meningioma, and nine proteins can be used to discriminate atypical from anaplastic meningiomas (57). Similar biomarkers were also reported in 2014; the expression of galectin-3, vimentin was decreased significantly in meningiomas, and the expression of 40S ribosomal protein S12 and glutathione S-transferase was increased significantly (59). It is worth mentioning that the function of galectin-3 was further investigated in 2017; high expression of galectin-3 was observed in meningioma infiltration and recurrence (60). However, the role of galectin-3 in meningioma remains controversial; there is still a need for further studies to confirm the exact mechanism of galectin-3 in meningioma. Recently, with highly sensitive instruments in proteomics, low-abundance proteins could be found to be meaningful in different grades of meningiomas. For example, comparative tissue proteomic analysis was performed by isobaric tags for relative and absolute quantification (ITRAQ)-based quantitative proteomics by using electrospray ionization-quadrupole-time of flight (ESI-Q-TOF) and thermo scientific Q exactive (Q-Exactive MS), which quantified many transmembrane receptors and transcription factors, such as activated RNA polymerase II transcriptional coactivator p15 in pathology of meningioma (61).

In addition, proteomics analysis has also been used to identify different subtypes of meningiomas. To explore the different protein expression patterns of bone-infiltrating and non-invasive meningioma, the researchers used a protein spectrum combined with surface-enhanced laser desorption/ionization time-of-flight mass spectrometry (SELDI), and the results show meaningful differences in fibrous and meningothelial grade I meningiomas that contribute to distinguish the two types of meningiomas (60). Therefore, invasive and non-invasive growth behavior of grade I fibrous and meningothelial meningioma can be distinguished by analyzing the protein profile of benign meningioma. Notably, the early diagnosis of invasive grade I meningioma is thought to contribute to follow-up policies and the issue of radiotherapy (62).

In addition to protein expression, proteomics studies about post-translational modifications have also been conducted to map the mechanisms of aggressiveness of meningiomas. By using two high-throughput technologies: unbiased iTRAQ LCMS/MS and biased Pamchip peptide arrays, it was found that the A-kinase anchor protein 12 (AKAP12) protein (a phosphoprotein) is downregulated in all grades of meningioma (58). Further studies have shown that knocking down AKAP12 in benign meningioma cells promotes proliferation, migration, and invasion, suggesting that AKAP12 is a central regulator of invasive meningioma progression (58). However, although studies have provided increasing evidence that post-translational modification is closely connected with cell-based functional characterization, which has a close connection with function and malignancy of the disease, phospho-proteomes are rarely studied in meningiomas (61, 63, 64).

## Multi-Omics Studies in Meningiomas

Despite a valuable contribution, the results from single omics are unable to map the comprehensive meningioma-related signaling pathways and networks. Therefore, advantages of integrated analysis using multi-omics data have been gradually revealed. For example, the *FoxM1* target gene in the case of increased *FoxM1* mRNA expression was identified by RNA sequencing, DNA methylation sequencing, and target gene expression profile from meningiomas with low survival rate and high local recurrence rate (65). In addition, integration of multi-omics data contributes to the identification of radiation-induced meningioma, an uncommon late risk of cranial irradiation with higher recurrence rate and pathologically malignant features compared to the sporadic meningioma (66). For example, comparative genome hybridization was used for the identification of chromosome 1p loss in radiation-induced meningioma (67). Notably, NF2 rearrangement in radiation-induced meningioma was identified through exome, methylation, and RNA-seq analysis from 31 cases, which can be used for the differentiation of radiation-induced meningioma from sporadic meningioma as neurofibromatosis type 2 (NF2) rearrangement has still not been reported in sporadic meningioma (68).

**Figure 4:**
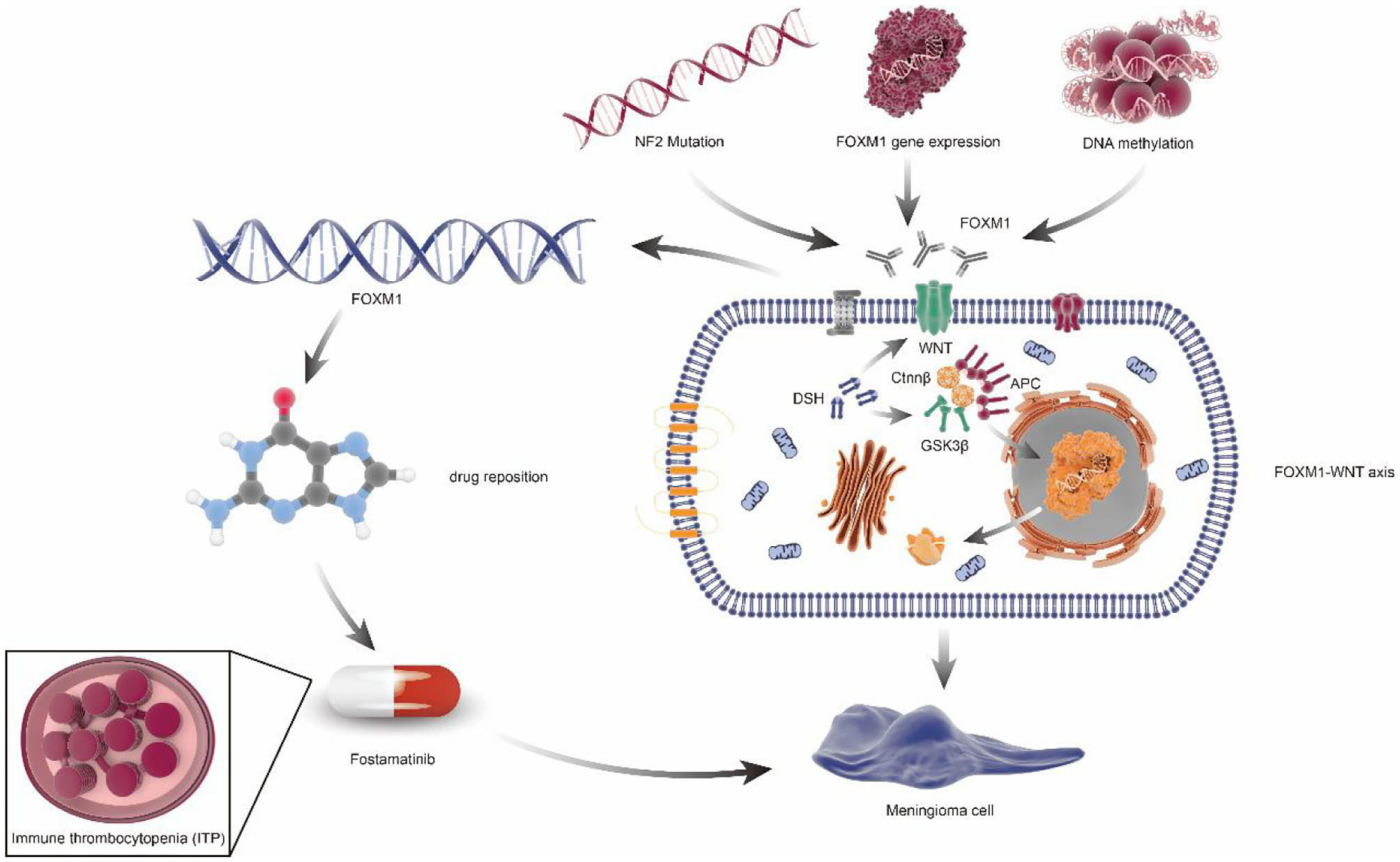
FOXM1/Wnt signaling axis drives meningioma prolife ratio and tumor growth. NF2 mutation, FOXM1 gene expression, and DNA methylation can cause the increase of FOXM1 expression or activity, which would activate the FOXM1/WNT signaling axis, resulting in primary or aggressive meningioma cell proliferation. In addition, through the principle of drug repositioning, fostamatinib, a kind of medicine aimed at chronic immune thrombocytopenia (ITP), which targets the FoxM1, may also be used in the treatment of meningiomas.

The target gene identified by multi-omics studies can potentially be used in drug repositioning in meningiomas (Supplementary Table 4), which appeared to be cheaper, quicker, and more effective (69). For example, Fostamatinib, targeting *FoxM1*, has been approved by the FDA for the treatment of chronic immune thrombocytopenia (ITP). Given the same putative drivers of disease associations, Fostamatinib may improve meningioma via regulating synthesis and secretion of tumor necrosis factor α(TNF-α) (70) (Figure 4).

## Conclusion

In 2016, the World Health Organization included the molecular standards into the classification of meningiomas (71). Soon after this, accurate pathological diagnosis and treatment decisions at the molecular level depend on powerful clinical molecular detection using genome, epigenome, and transcriptome tools is highly applied in clinical studies (72). Although it is necessary to carry out molecular detection of brain tumors in medicine, there are still great differences in the acquisition and utilization of molecular diagnosis technology in various institutions, and the lack of compensation for such detection is still a major obstacle (72). Notably, the important role of omics studies in the molecular level pathological study and grading of meningiomas has potential value in clinical diagnosis and treatment. Therefore, there is no doubt that multi-omics studies will shed further light on the novel strategies for the prediction, prevention, and treatment of meningiomas.

## Author Contributions

All authors listed have made a substantial, direct and intellectual contribution to the work, and approved it for publication.

## Conflict of Interest

The authors declare that the research was conducted in the absence of any commercial or financial relationships that could be construed as a potential conflict of interest.

## Supplementary Material

Supplementary Table 1 Summary of epigenomics studies for meningiomas.

Supplementary Table 2 Research on meningiomas in transcriptomics.

Supplementary Table 3 Proteomics research on meningiomas.

Supplementary Table 4 Meningioma driver genes-based drug-repositioning.

## Abbreviations

WHO: World Health Organization
GWAS: Genome Wide Association Study
UK: United Kingdom
aCGH: array comparative genomic hybridization
64-MMP: 64-CpG meningiomas methylation predictor
HRR: homologous recombination repair
PTX3: Recombinant Pentraxin 3
MN1: meningioma 1
miRNA: microRNA
snoRNA: small nucleolar RNA
sncRNA: small interfering RNA
siRNA: small interfering RNA
HMGN5: high-mobility group nucleosome-binding protein 5
K-Ras: K-rat sarcoma
SELDI-TOF MS: surface-enhanced laser desorption/ionization time-of-flight mass spectrometry
ITRAQ: isobaric tags for relative and absolute quantification
ESI-Q-TOF: electrospray ionization- quadrupole-time of flight
Q-Exactive MS: thermo scientific Q exactive
SELDI: surface-enhanced laser desorption/ionization time-of-flight mass spectrometry
AKAP12: a-kinase anchor protein 12
FoxM1: Forkhead Box M1
ITP: immune thrombocytopenia
TNF-α: tumor necrosis factor α
NF2: Neurofibromatosis Type 2.
